# Robust diagnosis and meta visualizations of plant diseases through deep neural architecture with explainable AI

**DOI:** 10.1038/s41598-024-64601-8

**Published:** 2024-06-13

**Authors:** Sasikaladevi Natarajan, Prasun Chakrabarti, Martin Margala

**Affiliations:** 1 Department of Computer Science and Engineering, School of Computing, SASTRA Deemed University, Thanjavur, TamilNadu 613401 India; 2 Sir Padampat Singhania University, Udaipur, Rajasthan India; 3 University of Louisiana at Lafayette, Lafayette, USA

**Keywords:** Plants, Deep neural networks, K-nearest neighbors, Plant disease classificaiton, Convolutional neural network, Explainable AI, Computational biology and bioinformatics, Plant sciences, Diseases, Engineering, Mathematics and computing

## Abstract

Deep learning has emerged as a highly effective and precise method for classifying images. The presence of plant diseases poses a significant threat to food security. However, accurately identifying these diseases in plants is challenging due to limited infrastructure and techniques. Fortunately, the recent advancements in deep learning within the field of computer vision have opened up new possibilities for diagnosing plant pathology. Detecting plant diseases at an early stage is crucial, and this research paper proposes a deep convolutional neural network model that can rapidly and accurately identify plant diseases. Given the minimal variation in image texture and color, deep learning techniques are essential for robust recognition. In this study, we introduce a deep, explainable neural architecture specifically designed for recognizing plant diseases. Fine-tuned deep convolutional neural network is designed by freezing the layers and adjusting the weights of learnable layers. By extracting deep features from a down sampled feature map of a fine-tuned neural network, we are able to classify these features using a customized K-Nearest Neighbors Algorithm. To train and validate our model, we utilize the largest standard plant village dataset, which consists of 38 classes. To evaluate the performance of our proposed system, we estimate specificity, sensitivity, accuracy, and AUC. The results demonstrate that our system achieves an impressive maximum validation accuracy of 99.95% and an AUC of 1, making it the most ideal and highest-performing approach compared to current state-of-the-art deep learning methods for automatically identifying plant diseases.

## Introduction

Modern agricultural techniques and tools have revolutionized food production, allowing us to meet the ever-growing demands of the world’s population. However, this progress is threatened by two major factors: climate change and plant disease. Climate change, as highlighted by Tai^1^, poses a significant risk to food production. Additionally, plant diseases, as noted by Strange^2^, also jeopardize our ability to meet global food requirements. The impact of plant diseases extends beyond the global scale; it has severe consequences for smallholder farmers who make up more than 80% of agricultural production^3^. Shockingly, half of the world’s hungry people reside in smallholder farmer households^4^. The presence of plant diseases can have a devastating effect on agricultural production, and if left undetected over time, it will exacerbate food insecurity^5^. To ensure a consistent food supply, it is crucial to detect and prevent plant diseases at an early stage. This task, however, presents challenges in rural regions of developing countries where farmers have limited access to agricultural experts. Therefore, there is a pressing need for automatic and efficient methods of detecting plant diseases in their early stages.

Different approaches, ranging from historical to modern, are utilized to prevent crop loss caused by plant diseases. Regardless of the specific methods employed^6^, the accurate and early identification of plant diseases is crucial for effective mitigation. The advancement of image classification has opened up new possibilities for applying computer vision research in the field of agriculture^7–9^. Deep learning models can now be employed to detect and classify plant diseases using images. This paper presents the development of a graph-based deep learning model that enables accurate and rapid diagnosis of plant diseases. Researchers have made significant advancements in the field of plant or leaf recognition by employing a range of autonomous systems. These systems often utilize deep learning and machine learning techniques to tackle this challenge effectively. To establish a robust classification system for plant diseases, it is imperative to train it on a comprehensive dataset that encompasses noisy images. Given the scarcity of manual professionals in densely populated countries like India, the development of an Artificial Intelligence-driven tool for identifying plants holds immense significance. The main contributions of this study can be summarized as follows:

- Deep Fused convolutional Neural Network and customized K-nearest Neighbor-based framework is proposed for the precise classification of plant diseases based on leaf images. This framework employs depthwise separable convolution to generate optimal feature maps on leaf images, enhancing multi-class classification.
- A dataset preparation pipeline is established for plant village dataset, facilitating the extraction of patches from high-density pixel images.
- Design of Meta agnostic model learning by Grad-CAM and Occulusion Sensitivity Analysis(OSA) to identify the parts of an image that influenced the model’s labeling decision
- The proposed framework thoroughly investigates the impact of fused features and customized KNN on classification performance, demonstrating that the model achieves the highest metrics in terms of accuracy, sensitivity, and specificity. The comprehensive investigation and comparative analysis underscore the efficacy of the proposed framework over other state-of-the-art methods.

The organization of this document is as follows. In Section “Related Work”, the most recent algorithms for machine learning and deep learning in the classification of plant diseases are presented. Section “Proposed HerbNet framework” introduces the HerbNet model, which is specifically designed for classifying the leaves of plant diseases. This section also provides a comprehensive description of the plant diseases used in this research. The experimental work and performance evaluation of the proposed system are elaborated in Section “Results and discussion”. Lastly, Section. “Conclusion” concludes the paper and discusses future directions.

## Related work

Plenty of researchers are currently exploring research-based image classification using medical images and satellite images. However, the classification of plant images based on leaves is still limited. In this study, my focus is on plant image classification using machine learning and deep learning techniques.

### Machine learning (ML) based techniques for plants disease identification

Aurangzeb et al.^10^ presented an automated model for detecting plant diseases in corn and potato leaves. The model utilized local ternary patterns (LTP), segmented fractal texture analysis (SFTA), and histogram-oriented gradient (HOG) as key features. To address the issue of high dimensionality, principal component analysis (PCA) and entropy skewness-based score values were used. While the system’s performance relies on the selected features, it may experience reduced accuracy in cases where an insufficient number of features are available. Durairaj et al.^11^ proposed a plant disease detection model that incorporates segmentation and an autoencoder technique to improve accuracy. The model began with noise removal through pre-processing, followed by segmentation using the Fuzzy C-means (FCM) algorithm. Features within the segmented area were then extracted using a discrete wavelet transform based on GLCM. However, the model’s drawback lies in its lower accuracy. Hossain et al.^12^ introduced a method based on color and texture, employing a KNN classifier for plant leaf disease detection and classification. Texture features were extracted from images of diseased leaves for classification purposes. The KNN Classifier was used to classify diseases such as canker, leaf spot, alternate, and bacterial blight, resulting in improved accuracy. However, the dataset used was relatively small.

Mahmoud et al.^13^ proposed a novel approach for classifying plant diseases using the PILAE algorithm and DCGAN. The PILAE algorithm trains a MLP neural network, while DCGAN performs feature extraction and generates synthetic samples for the minor classes. The classification is then performed using PILAE. However, this model has the drawback of requiring additional time for processing. Pardede et al.^14^ introduced an alternative unsupervised learning algorithm based on a convolutional autoencoder for plant disease recognition. The network first learns and identifies discriminative features without the need for data labelling. An SVM-based classifier is then used to classify the encoded features. This approach achieved better accuracy but with reduced robustness. Ramesh et al.^15^ proposed a machine learning approach for plant disease detection. They utilized a Random Forest classifier to identify infected and healthy leaves, and extracted features from images using the Histogram of Oriented Gradient (HOG) technique.

Shrivatsava et al.^16^ devised a rapid and efficient system to automatically identify and categorize diseases in rice plants. In this study, color played a crucial role, and a polynomial order-2 kernel function was employed in an SVM classifier for classification purposes. However, this system only considers a limited number of disease classes. In addition to an electron nose, a hybrid intelligent system incorporating SVM, probabilistic neural network (PNN), naïve Bayes, and fuzzy interference system was utilized for detection^17^. Furthermore, this system has been found to be time-consuming. Trang et al.^18^ introduced the use of an autoencoder as a feature extraction method for plant leaf disease detection, employing a dataset of leaf images. Initially, the original input leaf image was reconstructed using the encoder, and the features were extracted through the encoder. These extracted features were then fed into the SVM classifier for classification. Consequently, improved accuracy was achieved, albeit with increased computational processes. Table 1 lists out the ML based approaches for plant diseases classification with its limitation.

**Table 1:** State of the art machine learning approaches for plant disease classification and its performance analysis.

Author	Classifier	Data sets Used	Accuracy (%)	Limitations
^19^	SVM, KNN, CNN	Tomato leaf from Plant village data sets	SVM (88%), K-NN (97%), and CNN (99.6%)	More data set required
^16^	SVM	619 images from Indira Gandhi Agricultural University, Raipur	94.65	Need to enhance the detection performance
^20^	KNN	900 images from different regions of Himachal Pradesh and Uttarakhand	99	Experienced over- fitting problems
^21^	SVM, PNN, naïve Bayes, and fuzzy interference system	1000 Manually collected images of 10 species of herb leaves	SVM-97%, PNN-98%, NB- 94% SVM + PNN + NB-99%	More time complexity
^22^	M-SVM	137 grape leaf images from different regions like Pune, and Nasik	90.20	Only applicable for a particular disease and less performance
^10^	CSVM	Plant village data set	90	Very low detection accuracy
^23^	HAE	1350 images Scotnelson, Godliver, Plant village project, and real dataset of banana diseases	99.35	Only applicable for a particular disease and more complexity
^13^	PIlAE classifier	PlantVillage dataset, Swedish leaf dataset, Leafsnap dataset	100% (plant village data set), 99.32% (Swedish leaf dataset), 97.86% (Leaf snap dataset)	Time complexity
^18^	SVM	6477 images from the Plant village data set	98.8	Less performance
^11^	Autoencoder neural network	Plant village data set	80	Very low detection accuracy
^12^	KNN	237 leaf images from the Arkansas plant disease database and Reddit-plant leaf disease datasets	96.76	Need to analyze with a larger dataset
^14^	SVM	Plant village data set 54,309 images of 38 classes	87	Less robustness
^24^	SVM	150 images from Bangladesh Tea Research Institute	93	Less detection accuracy
^15^	Random forest	Created a data set of 160 images	70	Less performance, Need hybrid models
^25^	M-SVM	Plant Village (6212 images) and Citrus Images Database (670 images)	95.8	No proper diseased part and complexity were more
^26^	SVM	900 Images from cotton cultivation in the Buldhana district	83.26	Less accuracy and more computational complexity

### Deep learning (DL) based techniques for plants disease identification

Chohan et al.^27^ presented a highly effective neural network model to identify plant diseases. To increase the sample size, they initially performed data augmentation. Then, they applied a CNN with pooling and multiple convolution layers. As a result, the DL model achieved superior performance in detecting plant diseases. However, this model has two main drawbacks: overfitting issues and improved accuracy. Huang^28^ proposed a modified DL model for detecting and classifying grapevine diseases. In this DL model, they also utilized transfer learning with pre-trained machine learning approaches. The target diseases for classification and detection were phylloxera, measles, leaf blight, and black rot. Despite achieving maximum classification accuracy, the computation time was longer. Liu et al.^29^ introduced an enhanced deep convolutional neural network (DCNN) approach for efficient grape disease detection. They first analyzed the features of grape disease to improve the CNN. Then, they employed deep separable convolution to reduce the number of parameters and mitigate overfitting. The inception structure was utilized to enhance the capability of multiscale feature extraction. However, a limitation of this method is its lack of diversity.

Jun et al.^30^ proposed an enhanced v3 YOLO Convolutional neural network for the identification of tomato diseases. The dataset utilized in this study consists of tomato diseases and pests captured in a natural environment. Marino et al.^31^ introduced a novel DL model designed for the detection and localization of potato blemishes. Initially, the CNN was trained to categorize potato images, serving as a filter for selecting faces. Subsequently, an integrated SVM and autoencoder were applied to the chosen image to identify damage and greening disease in a patch-wise manner. However, this model is not suitable for real-time applications and is limited in its applicability to other plant diseases. Khamparia et al.^32^ developed a hybrid deep convolutional autoencoder network for the prediction and classification of seasonal crop diseases by combining a CNN and an autoencoder. The convolutional autoencoder network plays a crucial role in identifying crop diseases from the input crop image.

Ferentinos et al.^33^ introduced a CNN model to diagnose and detect plant diseases by utilizing images of both diseased plants and healthy leaves. Singth et al.^34^ developed a hybrid feature-based method for disease detection in plant leaves. This method combines color, texture, and deep features to form hybrid features, which are then classified using a random forest algorithm after data augmentation and Bayesian optimized support vector classifier. Lu et al.^35^ presented a deep CNN model specifically designed for automatically detecting and classifying rice diseases. The dataset used in their study consisted of 25 plant species with 58 distinct classes. However, this CNN model faces challenges such as class imbalance, overfitting, and exploding gradient. Despite these challenges, the deep CNN model proposed by Lu et al. effectively diagnoses ten types of rice diseases and includes multiple layers. Additionally, a tenfold cross-validation scheme was employed to enhance accuracy. To further improve the recognition rate, a Boltzmann machine and deep learning techniques were applied. However, it is important to note that a substantial amount of data is necessary to achieve better outcomes in this context.

Dhaka et al.^36^ conducted an extensive review of the current literature on the use of deep CNNs in predicting plant diseases from leaf images. The study provides valuable insights into pre-processing methods, CNN models, frameworks, optimization techniques, datasets, and performance measures, serving as a valuable reference for researchers navigating the complexities of selecting appropriate models in the agricultural sector. Wang et al.^37^ introduced the (Mobile Ghost Attention) MGA-YOLO algorithm for detecting apple diseases, which is a lightweight one-stage network. In this work, the authors replaced standard convolution with the ghost module to reduce the number of parameters and floating-point operations. They combined Mobile Inverted Residual bottleneck convolution with the YOLO network to enhance performance. Morbekar et al.^38^ evaluated YOLO-based algorithms for crop disease detection, achieving higher accuracy compared to other conventional models. This model can identify single or multiple infection regions on leaves. Among various deep learning algorithms, CNNs are the most widely used. Advanced methods like VGG 16, ResNet, and DenseNet have shown superior results. Additionally, Ferentinos^33^ achieved a remarkable accuracy of 99.53% using DL techniques. Table 2 lists out the DL based approaches for plant diseases classification with its limitation. Consequently, DL approaches continue to offer more effective solutions for detecting plant diseases compared to ML methods.

**Table 2:** State of the art deep learning approaches for plant disease classification and its performance analysis.

Author	Classifier	Data sets Used	Accuracy (%)	Limitations
^39^	Yolo v5	Money Plant Leaves	93	Incorrect detections, suggesting the need for further refinement of the model’s mechanism
^40^	Inception	Plant Village dataset	99.95	Identifying the optimal parameters is challenging
^23^	Hybrid model with Bayesian optimized support vector machine	Plant Village data sets	96.1	Execution time high
^41^	VGG-19 based Transfer learning	Kaggle	96.08	Datasets of rice disease, which makes the problem more complex. Overfitting and under fitting
^42^	14-DCNN	Open data repositories	99.96	Time consuming
^43^	Xception, DenseNet	6 plant species from Kaggle	96.9%	Datasets were not used to analyze the performance
^44^	Two-stage Convolutional Neural Network	Citrus data sets from Kaggle	94.27	Execution time was more
^30^	Inception	Public available data sets	97.22	Less diversity
^28^	Convolutional Neural Network model, VGG 16, AlexNet and MobileNet	Plant village data set	98, 77, 97	It takes more computation time
^32^	Hybrid deep convolutional autoencoder network	Plant village data sets	97.50	Used small dataset
^45^	ResNet34	Plant Village dataset	97.2	Complexity high
^31^	DL model	Manually created data set with a black background	93	Only used for potato disease
^46^	AlexNet, GoogleNet Inception structure	Manually collected data sets	97.62	High-quality images were not considered to detect the diseases accurately and timely
^33^	AlexNetOWTBn, VGG	Open Database of 25 plants	99.53	Time demanding
^47^	LeNet	14 crop images from Kaggle	94 0.95	A large number of training data is required
^35^	CNN	Manually collected data sets	94.48	Identifying the optimal parameters was challenging

This section extensively explores deep learning and machine learning models for diagnosing plant diseases. The deep learning model exhibits superior accuracy when compared to the machine learning model. Existing literature indicates that the current deep learning models for plant disease detection are based on pre-trained deep convolutional neural networks, which have been trained using benchmark color images. However, fine-tuning or applying transfer learning to these pre-trained neural networks can result in accuracy issues for certain plant classes. Consequently, there is a growing demand for novel deep convolutional neural networks specifically designed for different plant datasets. The need of the hour is a unique model that can effectively detect various types of plant diseases. This paper proposes a deep convolutional neural architecture that caters to the detection of all kinds of plant diseases.

## Proposed HerbNet framework

**Figure 1:**
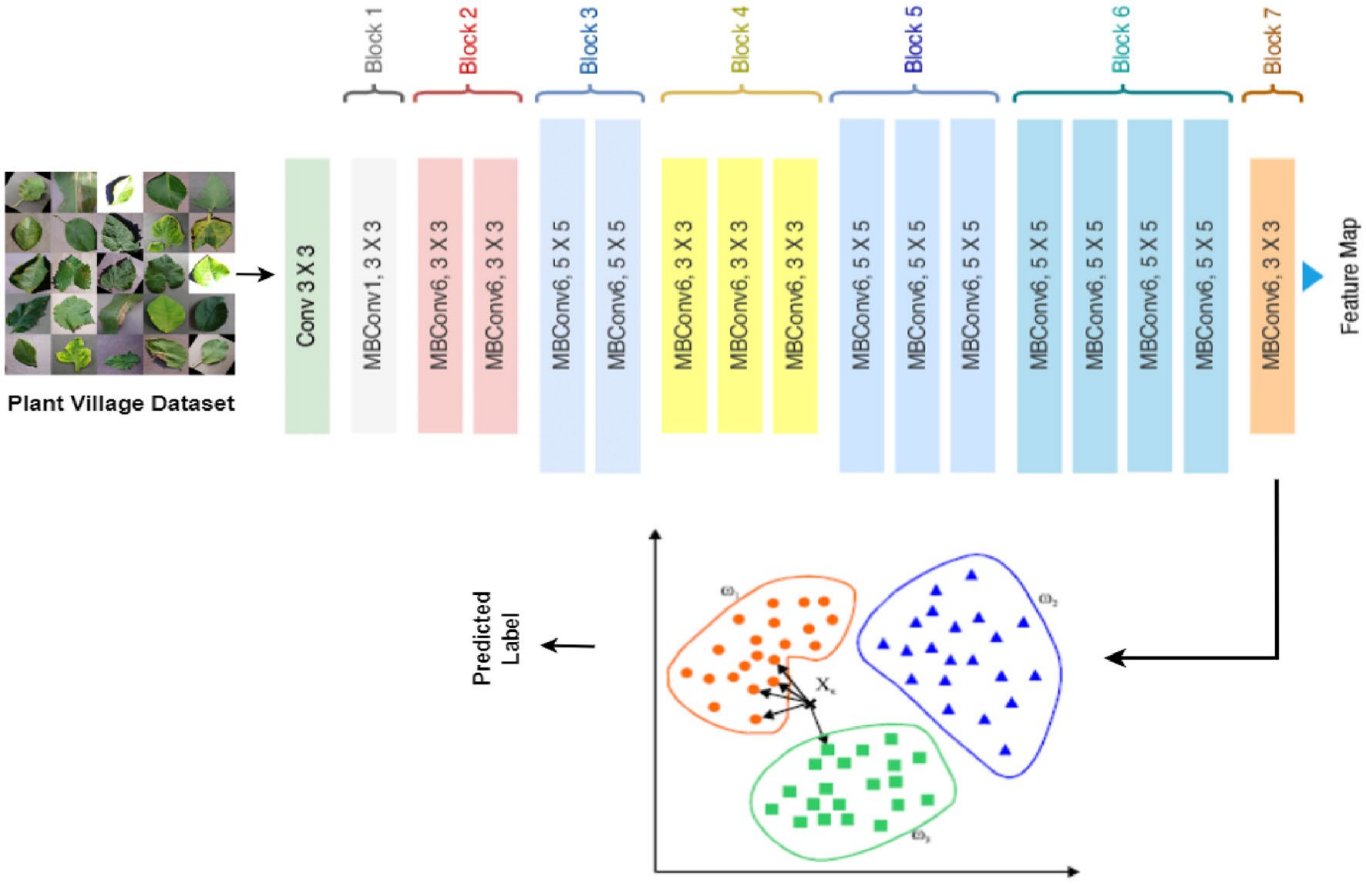
Proposed HerbNet Framework for plant disease classification.

The proposed deep learning framework comprises of two main phases: the model development phase and the meta-visualization phase. The model development phase involves a three-step process which includes feature extraction, training, and validation. In order to carry out the training and validation, a dataset containing plant leaves is utilized. Figure 1 illustrates the proposed framework for identifying plant diseases.

The images provided are in jpeg format and possess a resolution of 1600*1200. Subsequently, these images undergo resizing to 224×224 utilizing the red, green, and blue channels. The dataset is divided into 70% for training purposes and 30% for validation.

### Deep feature extraction

**Figure 2:**
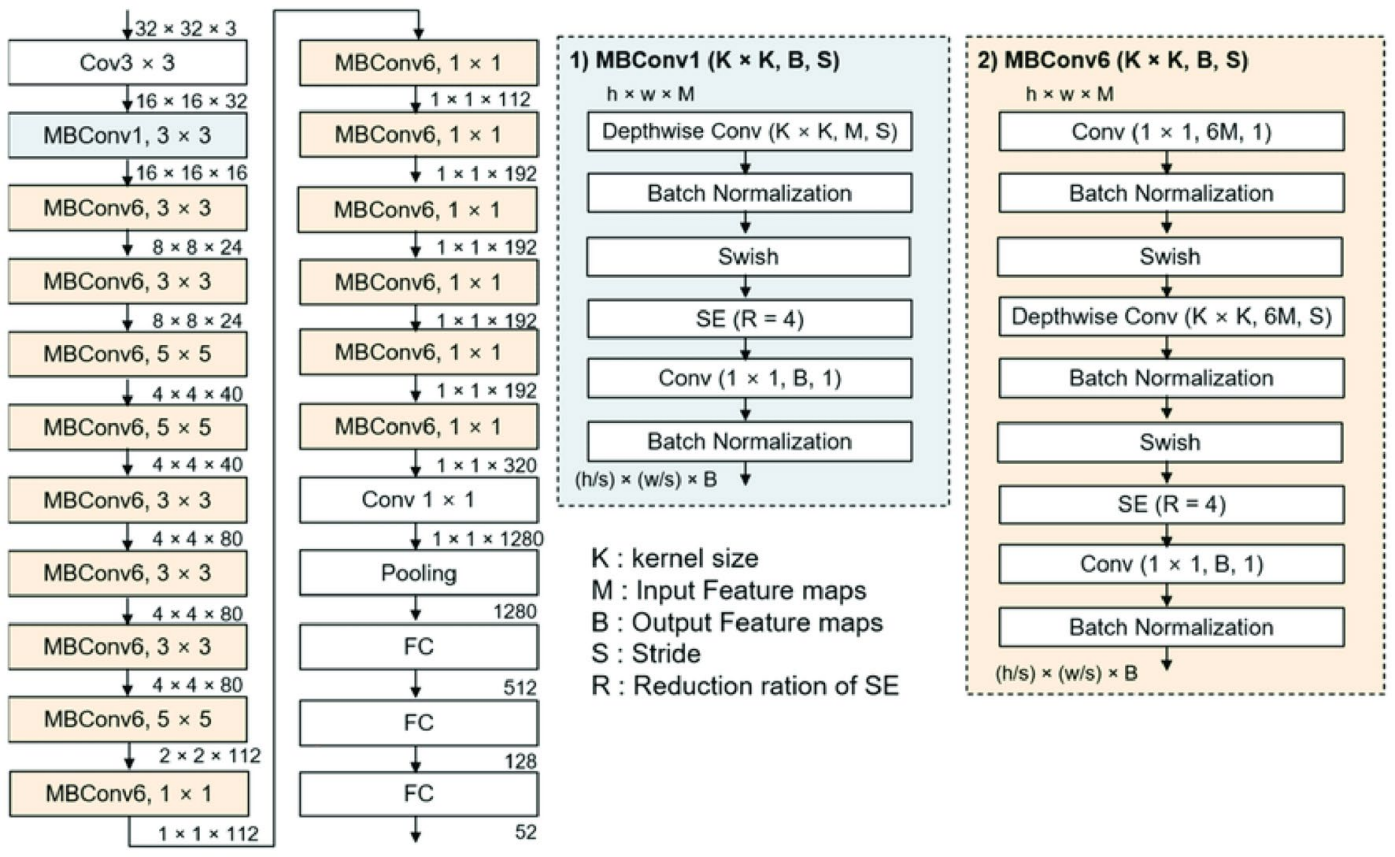
EfficientNetB0 architecture with the layers details (Tan, 2019).

Deep Convolutional Neural Networks (DCNN) have made remarkable advancements in image analysis, surpassing conventional machine learning models in feature extraction, classification, and segmentation. DCNNs excel at extracting both low-level and high-level features, where the initial layers focus on low-level features and the upper layers’ capture complex high-level features. These extracted features can be utilized for classification using either deep classifiers or traditional classifiers. To extract intricate high-level features from images, the EfficientNetB0 architecture is employed. Unlike densely packed neural network architectures, this architecture implements consistent compound scaling across all image dimensions. The pooling layer further extracts deep and condensed features. With a mere 5.3 million trainable parameters, this architecture significantly reduces training time compared to other densely packed architectures. The input layers accept RGB images of size 224 × 224. By leveraging this network, the proposed architecture effectively extracts features from images, resulting in an output of a feature vector with a size of 1280 from the pooling layer. The feature extraction phase comprises of 16 Mobile Inverted Bottleneck Convolutional Layers (MBConv) with kernel sizes of 3 × 3 or 5 × 5, as depicted in Fig. 2.

### KNN for classification

K-nearest Neighbor (KNN) is a supervised classification algorithm that utilizes the concept of finding similarities between data points to make predictions. It is commonly employed for classification tasks, although it can also be applied to regression problems. The fundamental assumption of KNN is that the data points either form clusters or are located in close proximity to each other. Notably, KNN is a non-parametric algorithm that refrains from making any assumptions about the data. Multiclass classification is considered for solving the proposed problem. Consider $N$, the total number of leaf images and ${N}_{i}$ the number of images in each class $i$ where $i=\text{1,2},..38$.

$$p\left(x\right)=\frac{k}{NV(x)}$$

where, $V\left(x\right)$ is the multidimensional feature space with radius being the distance to the k-nearest samples. Then compute,

$$p\left(x,{C}_{i}\right)=\frac{{k}_{i}}{NV(x)}$$

Compute the posterior probability as,

$$p\left({C}_{i}|x\right)=\frac{p\left(x,{C}_{i}\right)}{p(x)}=\frac{{k}_{i}}{k}$$

### Explainable AI for meta visualizations

The use of an explainable AI not only provides high accuracy, but also provides physiological insight into model predictions, thus generating confidence in model predictions. These explained predictions lend themselves for eventual use in precision agriculture and research application using automated plant diseases classification. In this work, the gradient based and occlusion based explainable AI techniques are developed for meta visualization of plant diseases classification.

#### Gradient based visualization

The Grad-CAM approach uses gradient information from the Convolutional Neural Network’s (CNN) final convolutional layer to assign meaningful values to each neuron. We compute the gradient score for the class ${y}^{c}$ based on feature map activations ${A}^{k}$ of a convolutional layer to compute the class-discriminative localization map Grad-CAM of width $u$ and height $v$ for a class $c$. The neuron significance weight is calculated based on Eq. 1.

1 $${\alpha }_{k}^{c}=\frac{1}{Z}\sum_{i}\sum_{i}\frac{\partial {y}^{c}}{\partial {A}_{ij}^{k}}$$

This weight ${a}_{k}^{c}$ is a partial linearization of the deep network downstream from A that captures the significance of feature map k for a target class c. As a result, we execute a weighted combination of forward activation maps to get the Grad-CAM of class $c$, as shown in Eq. 2.

2 $${L}_{Grad-CAM}^{c}=ReLU\left(\sum_{k}{\alpha }_{k}^{c}{A}^{k}\right)$$

Our exclusive attention to the qualities that favorably influence the class of interest motivates the use of a Rectified Linear Unit (ReLU) to the linear combination of maps. Specifically, we aim to enhance the intensity of pixels that contribute to the increase of ${y}^{c}$. Conversely, pixels with negative values are more likely to pertain to other categories within the image.

#### Occlusion sensitivity based network interpretation

We have utilized the occlusion sensitivity analysis (OSA), an efficient perturbation-based technique. This approach first quantifies the little variations in the class score by excluding various areas of the input image and making modest adjustments. The next step is to combine the differences in score for each region to create a saliency map for the input image, also known as a sensitivity map. The local image areas in this sensitivity map that show much variance are emphasized as the factors that positively affect the class score. Consequently, the occlusion sensitivity map offers insightful information about the picture characteristics that influence the final judgment and highlights the causes of the network’s classification failure. Meaningful Perturbation and Extremal Perturbation produce an opposite-natured saliency map from the OSA. These techniques preserve the components or areas that contribute significantly while hiding the components or regions that contribute less.

Let ${I}_{t}$ be the test image with size $H\times W\times 3$ where $H$ is the height, and $W$ is the width of red, blue, and green color channels. The first step generates the occlusion masks Ω based on the trained image set. An occlusion mask represents the movement of an anchor point or an object. Applying an occlusion mask $\Omega$ i causes a considerable change in the output score, and the relevant occluded regions are shown as output. Measurements of the co-occurrence between occlusion masks may be made using various methods. We gauge co-occurrence based on the displacement vectors’ fluctuation pattern as determined by optical flow. We define the co-occurrence between the occlusion masks ${\Omega }_{i, }{\Omega }_{j}$ as represented in Eq. 3.

3 $$C\left({\Omega }_{i, }{\Omega }_{j}\right)=\frac{{v}_{i} . {v}_{j}}{\Vert {v}_{i}\Vert \Vert {v}_{j}\Vert }$$

where, $${v}_{i}={\left[{({a}_{i}^{2}-{a}_{i}^{1})}^{T},\dots ,{({a}_{i}^{T}-{a}_{i}^{T-1})}^{T}\right]}^{T}$$,

${v}_{j}={\left[{({a}_{j}^{2}-{a}_{j}^{1})}^{T},\dots ,{({a}_{j}^{T}-{a}_{j}^{T-1})}^{T}\right]}^{T}$ for each trained image set anchor point.

Mask integration: We combine occlusion masks based on co-occurrence. We combine the *K* highly co-occurring masks with the i^th^ mask Ω_*i*_ . In order to construct the integration mask *i* and ensure that the masks obscure all target regions, we apply the element-wise product. The output score of the target class is then calculated after feeding the occluded test picture into a classification algorithm. If the output score drops, we assume that the occluded regions are crucial for the categorization. The weighted total of the mask for the visualization map $S$ is as shown in Eq. 4.

4 $$S=\frac{1}{N}\sum_{i=1}^{N}f({\Omega }_{i} V).{\Omega }_{i}$$

where *f* is a model for deep classification.

## Results and discussion

The model was implemented using the MATLAB software^48^ with version 2020b on a workstation that had an NVIDIA GPU and 16GB RAM. For this study, the researchers selected the Plant village dataset as the benchmark. The same dataset was used to both train and evaluate the proposed framework. This dataset consists of 1,63,000 images that are divided into 38 classes, making it an unbalanced dataset.

### Performance analysis of feature extraction phase

The deep neural network has been optimized to efficiently extract features from the pooling layer. The primary goal of the pooling layers is to reduce the dimensions of the hidden layer by combining the outputs of neuron clusters from the previous layer into a single neuron. This reduction in size effectively decreases the number of parameters that need to be learned and reduces the computational workload within the network. Furthermore, the pooling layer effectively summarizes the characteristics produced by a convolution layer within a specific region of the feature map.

**Figure 3:**
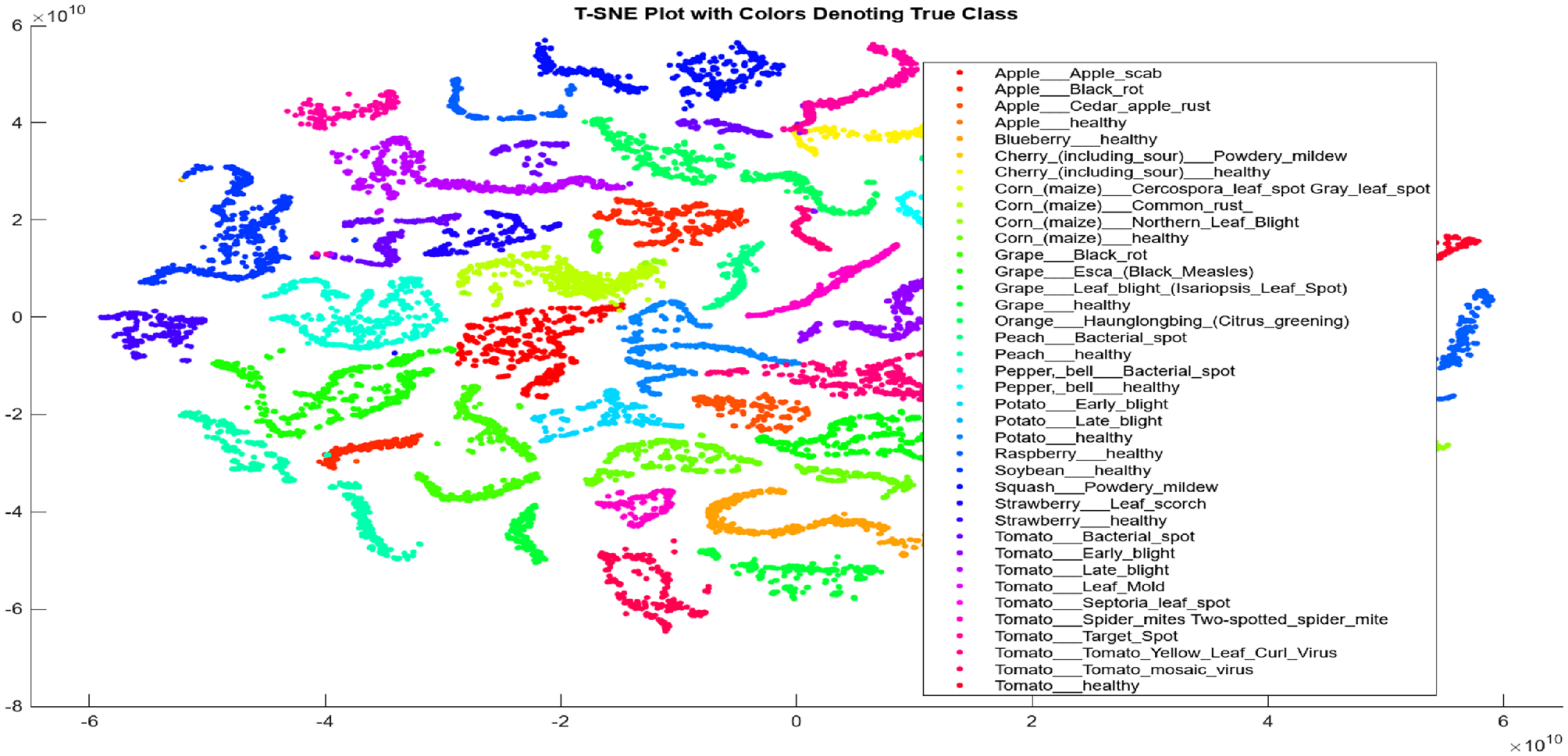
t-SNE plot of the features extracted from each class of plant disease images.

Figure 3 presents the t-SNE plot, which showcases the extracted features (25 features alone for improved visualization) for all 30 classes. One significant advantage of t-SNE is its ability to preserve the local structure. In other words, data points that are close to each other in the high-dimensional dataset will appear close to each other in the chart. Additionally, t-SNE generates visually appealing visualizations.

### Performance analysis of classification phase

**Figure 4:**
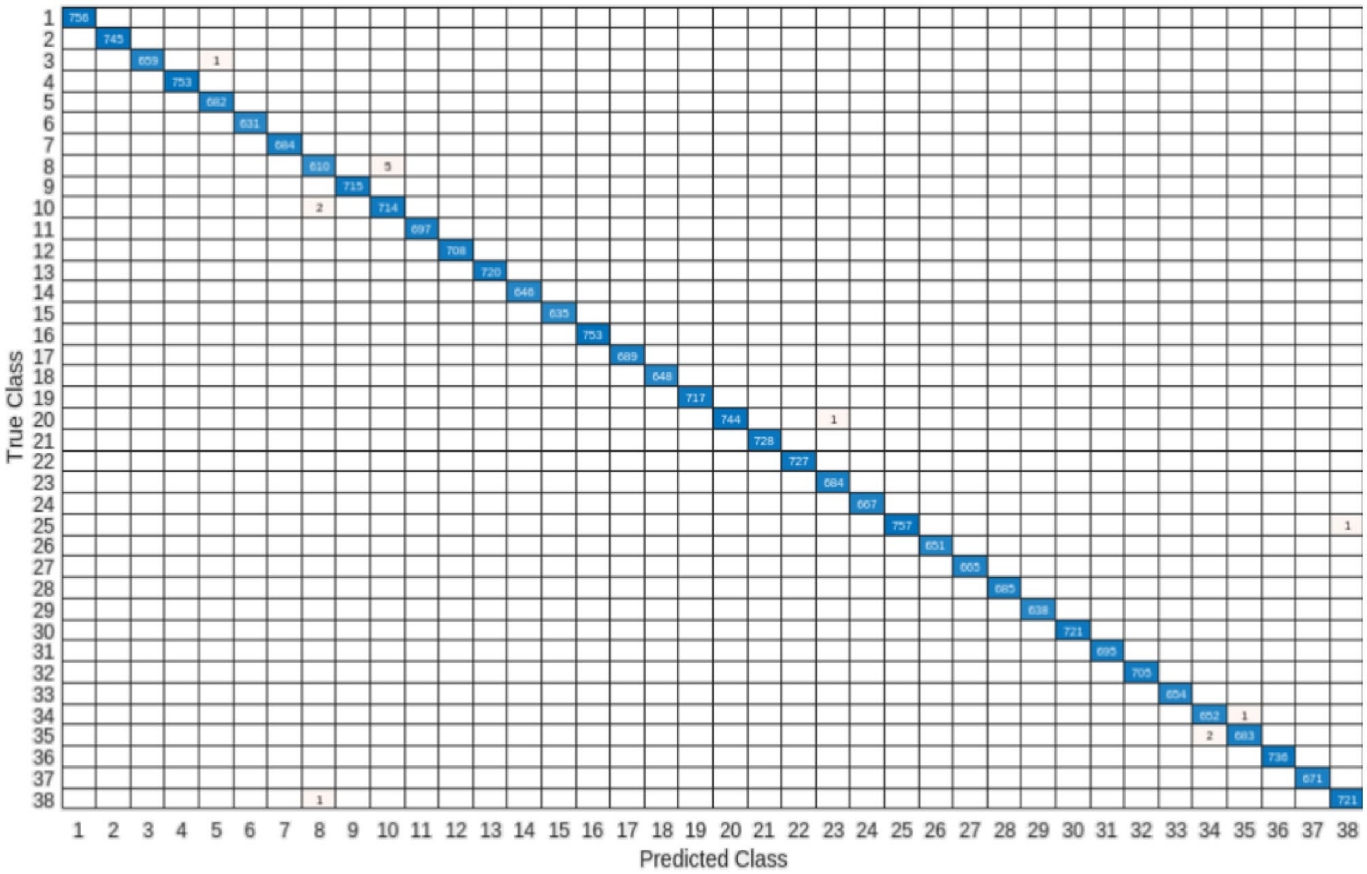
HerbNet- Confusion Matrix for all 38 classes of plant disease images.

**Figure 5:**
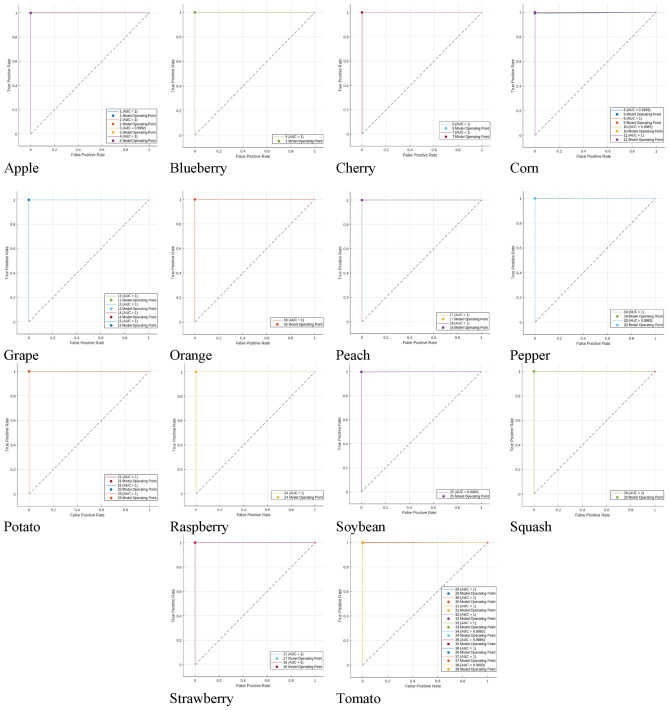
HerbNet- RoC plot for each type of plants.

Customized KNN classification attains the utmost level of precision. A summary in matrix form is presented in Fig. 4, which exhibits a confusion chart. This chart visually represents the count of accurate and inaccurate predictions for each label. Remarkably, it demonstrates a 100% accuracy rate across all labels. The overall performance of the method is evaluated using the ROC curve. This curve aids in determining the optimal cut-off value for assessing the presence of each label. The ROC plot of the proposed model is showcased in Fig. 5. The ROC curve is a probability curve, and the AUC (Area Under the Curve) signifies the model’s capability to differentiate between classes. Figure 5 reveals that the AUC is optimal for each label.

**Figure 6:**
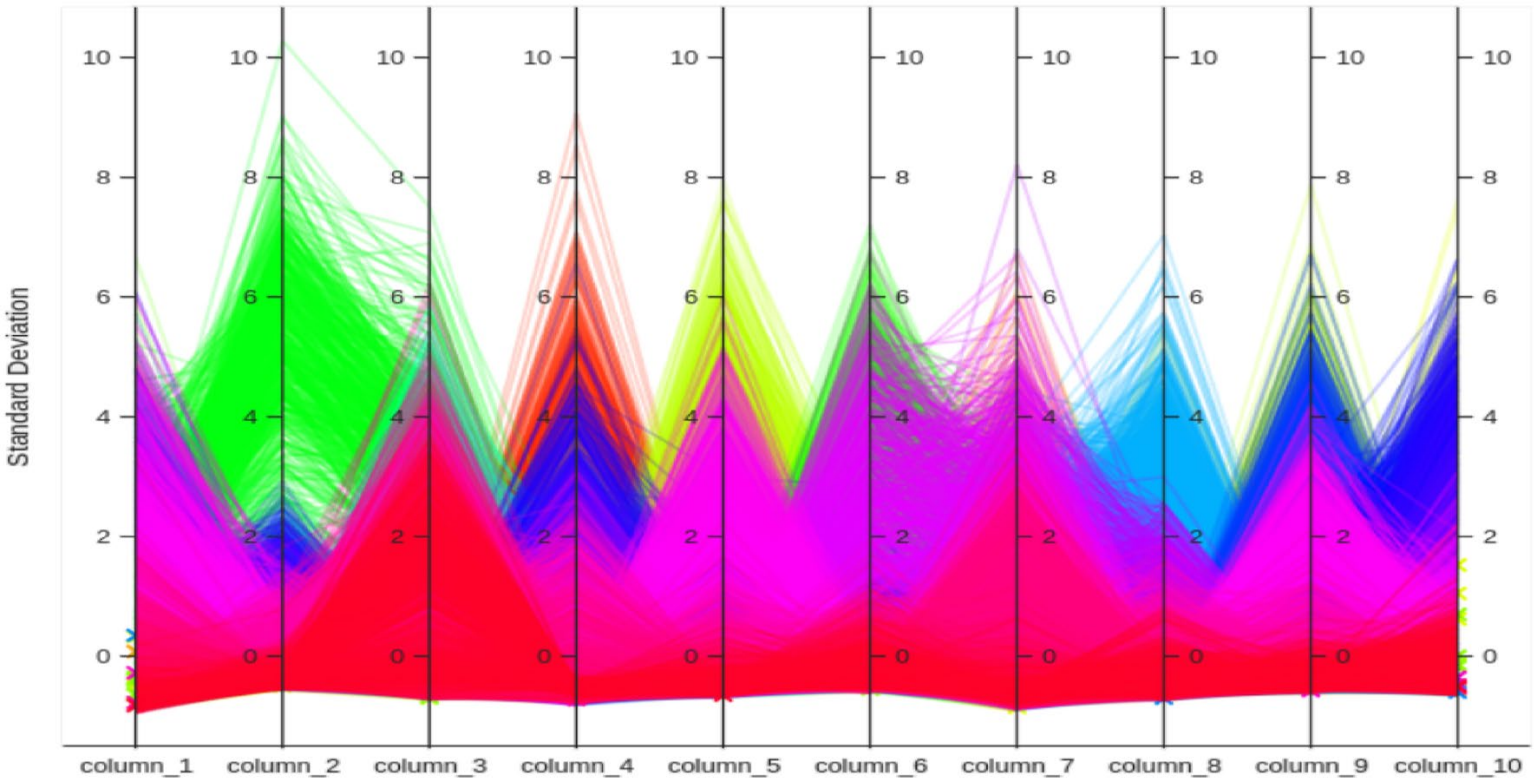
Parallel coordinate plot for the first 10 features of all 38 classes of images.

In order to create the curve, it is necessary to plot the False Positive Rate (FPR) against the True Positive Rate (TRP). The model’s effectiveness can be assessed by calculating the Area Under Cover, which is obtained from the ROC. The parallel plot, also known as the parallel coordinates plot, allows for the comparison of characteristics across multiple individual observations using numerical variables. Each vertical barrepresents a variable and typically has its own scale. This concept is illustrated in Fig. 6.

### Performance analysis of the visualization phase

**Figure 7:**
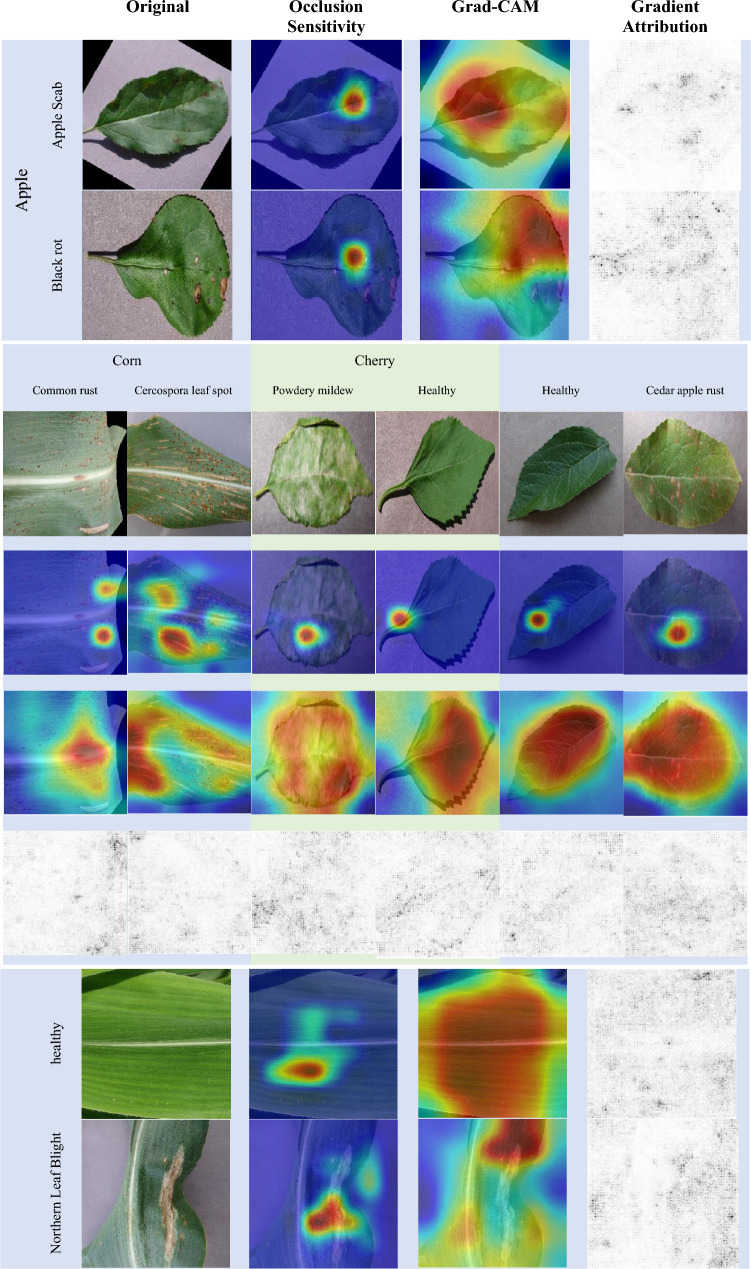
HerbNet Meta Visualizations of Pooling features of Apple, corn and cherry sub classes.

The occlusion sensitivity technique generates a visual representation that shows how the classification score for a specific target class changes when certain parts of the input image are concealed using a mask. This is shown in Fig. 7. On the other hand, Grad-CAM uses the gradient of the classification score for the convolutional features identified by the network to identify the essential areas of the image for classification. This is depicted in Fig. 7. This technique of gradient attribution produces maps that highlight the pixels that are most important for the network’s classification.

### Comparative analysis with other plant diseases classification models

In this section, the performance of HerbNet is assessed by comparing it with other existing methods for categorizing plant diseases. The Mendeley dataset is utilized to train and validate HerbNet, and the deep learning model constructed on the same dataset is contrasted with the suggested model in terms of training and validation performance. Recently, researchers have devised various alternative approaches to identify plant diseases using leaf photographs. The comparison results of the suggested model, including accuracy and other performance parameters, are presented in Table 3. Table 3 demonstrates that, in comparison to all other SOTA models, the proposed model HerbNet showcases an impressive validation accuracy of 99.95%. This unequivocally indicates that the preliminary model outperforms all existing state-of-the-art models in the classification of plant diseases.

**Table 3:** Comparative analysis of the proposed model with other State of the art models for plant disease classsifications.

Author	Classifier	Accuracy (%)
^28^	Convolutional Neural Network model- AlexNet	77.00
^11^	Autoencoder neural network	80.00
^14^	SVM	87.00
^10^	CSVM	90.00
^25^	M-SVM	95.80
^41^	VGG-19 based Transfer learning	96.08
^34^	Hybrid model with Bayesian optimized support vector machine	96.10
^28^	Convolutional Neural Network model- MobileNet	97.00
^45^	ResNet34	97.20
^29^	Inception	97.22
^32^	Hybrid deep convolutional autoencoder network	97.50
^28^	Convolutional Neural Network model—VGG 16	98.00
**Proposed**	**Customized KNN**	**99.95**

## Conclusion

In this research, we present a novel neural architecture that is specifically designed to identify plant diseases in a deep and explainable manner. Our approach involves extracting deep features from a down sampled feature map of a fine-tuned neural network. These features are then classified using a customized K-Nearest Neighbors Algorithm. To ensure the effectiveness of our model, we train and validate it using the largest standard plant village dataset, which comprises 38 classes. In order to assess the performance of our proposed system, we measure specificity, sensitivity, accuracy, and AUC. The findings reveal that our system achieves an outstanding maximum validation accuracy of 99.95% and an AUC of 1. This establishes our approach as the most optimal and highest-performing method when compared to current state-of-the-art deep learning techniques for automatically identifying plant diseases. In future, the proposed model will be integrated as mobile app with real time images to assist the farmers to identify the plant diseases in the affordable and efficient manner.

## Ethical approval

The above work is not based on animal or real-time human data. It is based on the data from the public repository.

## Data Availability

This work is based on the plant village dataset. It is publicly available at https://www.kaggle.com/datasets/abdallahalidev/plantvillage-dataset

## References

[1] Tai AP, Martin MV, Heald CL (2014). The threat to future global food security from climate change and ozone air pollution. Nat. Clim. Change.

[2] Strange RN, Scott PR (2005). Plant disease: A threat to global food security. Annu. Rev. Phytopathol..

[3] UNEP. Smallholders, food security, and the environment. Rome: International Fund for Agricultural Development (2013).

[4] Sanchez PA, Swaminathan MS (2005). Cutting world hunger in half. Science.

[5] Faithpraise F, Birch P, Young R, Obu J, Faithpraise B, Chatwin C (2013). Automatic plant pest detection and recognition using k-means clustering algorithm and correspondence filters. Int. J. Adv. Biotechnol. Res..

[6] Ehler LE (2006). Integrated pest management (IPM): Definition, historical development and implementation, and the other IPM. Pest Manag. Sci..

[7] Mohanty SP, Hughes DP, Salathé M (2016). Using deep learning for image-based plant disease detection. Front. Plant Sci..

[8] Sladojevic S, Arsenovic M, Anderla A, Culibrk D, Stefanovic D (2016). Deep neural networks-based recognition of plant diseases by leaf image classification. Comput. Intell. Neurosci..

[9] Reyes AK, Caicedo JC, Camargo JE (2015). Fine-tuning deep convolutional networks for plant recognition. CLEF (Working Notes).

[10] Aurangzeb K, Akmal F, Khan MA, Sharif M, Javed MY, Aurangzeb K (2020). Advanced machine learning algorithm based system for crops leaf diseases recognition. 2020 6th Conference on Data Science and Machine Learning Applications (CDMA).

[11] Durairaj V, Surianarayanan C (2020). Disease detection in plant leaves using segmentation and autoencoder techniques. Malaya J..

[12] Hossain E, Hossain MF, Rahaman MA, Hossain E, Hossain MF, Rahaman MA (2019). A color and texture based approach for the detection and classification of plant leaf disease using KNN classifier. 2019 international conference on electrical, computer and communication engineering (ECCE).

[13] Mahmoud MA, Guo P, Wang K (2020). Pseudoinverse learning autoencoder with DCGAN for plant diseases classification. Multimed. Tools Appl..

[14] Pardede HF, Suryawati E, Sustika R, Zilvan V, Pardede HF, Suryawati E, Sustika R, Zilvan V (2018). Unsupervised convolutional autoencoder-based feature learning for automatic detection of plant diseases. 2018 international conference on computer, control, informatics and its applications (IC3INA).

[15] Ramesh S, Hebbar R, Niveditha M, Pooja R, Shashank N, Vinod PV, Ramesh S, Hebbar R, Niveditha M, Pooja R, Shashank N, Vinod PV (2018). Plant disease detection using machine learning. 2018 International conference on design innovations for 3Cs compute communicate control (ICDI3C).

[16] Shrivastava VK, Pradhan MK (2021). Rice plant disease classification using color features: A machine learning paradigm. J. Plant Pathol..

[17] Pravin Kumar SK, Sumithra MG, Saranya N (2021). Particle swarm optimization (PSO) with fuzzy c means (PSO-FCM)–based segmentation and machine learning classifier for leaf diseases prediction. Concurr. Comput. Pract. Exp..

[18] Trang K, TonThat L, Thao NGM, Trang K, TonThat L, Thao NGM (2020). Plant leaf disease identification by deep convolutional autoencoder as a feature extraction approach. 2020 17th International Conference on Electrical Engineering/Electronics, Computer, Telecommunications and Information Technology (ECTI-CON).

[19] Harakannanavar SS, Rudagi JM, Puranikmath VI, Siddiqua A, Pramodhini R (2022). Plant leaf disease detection using computer vision and machine learning algorithms. Glob. Transit. Proc..

[20] Singh S, Gupta S, Tanta A, Gupta R (2022). Extraction of multiple diseases in apple leaf using machine learning. Int. J. Image Grap..

[21] Mustafa MS, Husin Z, Tan WK, Mavi MF, Farook RSM (2020). Development of automated hybrid intelligent system for herbs plant classification and early herbs plant disease detection. Neural Comput. Appl..

[22] Jadhav SB, Udupi VR, Patil SB (2021). Identification of plant diseases using convolutional neural networks. Int. J. Inf. Technol..

[23] Singh V, Sharma N, Singh S (2020). A review of imaging techniques for plant disease detection. Artif. Intell. Agric..

[24] Hossain S, Mou RM, Hasan MM, Chakraborty S, Razzak MA, Hossain S, Mou RM, Hasan MM, Chakraborty S, Razzak MA (2018). Recognition and detection of tea leaf’s diseases using support vector machine. 2018 IEEE 14th International Colloquium on Signal Processing & Its Applications (CSPA).

[25] Sharif M, Khan MA, Iqbal Z, Azam MF, Lali MIU, Javed MY (2018). Detection and classification of citrus diseases in agriculture based on optimized weighted segmentation and feature selection. Comput. Electron. Agric..

[26] Sarangdhar AA, Pawar VR, Sarangdhar AA, Pawar VR (2017). Machine learning regression technique for cotton leaf disease detection and controlling using IoT. 2017 International Conference of Electronics, Communication and Aerospace Technology (ICECA).

[27] Chohan M, Khan A, Chohan R, Katpar SH, Mahar MS (2020). Plant disease detection using deep learning. Int. J. Recent Technol. Eng..

[28] Huang, Z., Qin, A., Lu, J., Menon, A., & Gao, J. (2020, November). Grape leaf disease detection and classification using machine learning. In *2020 international conferences on internet of things (iThings) and IEEE green computing and communications (GreenCom) and IEEE cyber, physical and social computing (CPSCom) and IEEE smart data (SmartData) and IEEE congress on Cybermatics (Cybermatics)* (pp. 870–877). IEEE.

[29] Liu B, Tan C, Li S, He J, Wang H (2020). A data augmentation method based on generative adversarial networks for grape leaf disease identification. IEEE Access.

[30] Liu J, Wang X (2020). Tomato diseases and pests detection based on improved Yolo V3 convolutional neural network. Front. Plant Sci..

[31] Marino S, Beauseroy P, Smolarz A (2019). Deep learning-based method for classifying and localizing potato blemishes. ICPRAM.

[32] Khamparia A, Saini G, Gupta D, Khanna A, Tiwari S, de Albuquerque VHC (2020). Seasonal crops disease prediction and classification using deep convolutional encoder network. Circuits Syst. Signal Process..

[33] Ferentinos KP (2018). Deep learning models for plant disease detection and diagnosis. Comput. Electron. Agric..

[34] Singh AK, Sreenivasu SVN, Mahalaxmi USBK, Sharma H, Patil DD, Asenso E (2022). Hybrid feature-based disease detection in plant leaf using convolutional neural network, bayesian optimized SVM, and random forest classifier. J. Food Qual..

[35] Lu Y, Yi S, Zeng N, Liu Y, Zhang Y (2017). Identification of rice diseases using deep convolutional neural networks. Neurocomputing.

[36] Dhaka VS, Meena SV, Rani G, Sinwar D, Ijaz MF, Woźniak M (2021). A survey of deep convolutional neural networks applied for prediction of plant leaf diseases. Sensors.

[37] Wang Y, Wang Y, Zhao J (2022). MGA-YOLO: A lightweight one-stage network for apple leaf disease detection. Front. Plant Sci..

[38] Morbekar A, Parihar A, Jadhav R, Morbekar A, Parihar A, Jadhav R (2020). Crop disease detection using YOLO. 2020 International Conference For Emerging Technology (INCET).

[39] Khalid M, Sarfraz MS, Iqbal U, Aftab MU, Niedbała G, Rauf HT (2023). Real-time plant health detection using deep convolutional neural networks. Agriculture.

[40] Shoaib M, Shah B, Ullah I, Ali F, Park SH (2022). Deep learning-based segmentation and classification of leaf images for detection of tomato plant disease. Front. Plant Sci..

[41] Latif G, Abdelhamid SE, Mallouhy RE, Alghazo J, Kazimi ZA (2022). Deep learning utilization in agriculture: Detection of rice plant diseases using an improved CNN model. Plants.

[42] Pandian JA, Kumar VD, Geman O, Hnatiuc M, Arif M, Kanchanadevi K (2022). Plant disease detection using deep convolutional neural network. Appl. Sci..

[43] Kabir, M. M., Ohi, A. Q., & Mridha, M. F. (2021). A multi-plant disease diagnosis method using convolutional neural network. *Computer vision and machine learning in agriculture*, 99–111.

[44] Syed-Ab-Rahman SF, Hesamian MH, Prasad M (2022). Citrus disease detection and classification using end-to-end anchor-based deep learning model. Appl. Intell..

[45] Harte Emma, Plant Disease Detection using CNN, September 2020, 10.131 40/RG.2.2.36485.99048.

[46] Chao X, Sun G, Zhao H, Li M, He D (2020). Identification of apple tree leaf diseases based on deep learning models. Symmetry.

[47] Kaushik M, Prakash P, Ajay R, Veni S, Kaushik M, Prakash P, Ajay R, Veni S (2020). Tomato leaf disease detection using convolutional neural network with data augmentation. 2020 5th International Conference on Communication and Electronics Systems (ICCES).

[48] https://ww2.mathworks.cn/en/products/

